# A Novel Lactococcal Vaccine Expressing a Peptide from the M2 Antigen of H5N2 Highly Pathogenic Avian Influenza A Virus Prolongs Survival of Vaccinated Chickens

**DOI:** 10.1155/2013/316926

**Published:** 2013-05-22

**Authors:** Kaleb A. Reese, Christopher Lupfer, Rudd C. Johnson, Georgi M. Mitev, Valerie M. Mullen, Bruce L. Geller, Manoj Pastey

**Affiliations:** ^1^Department of Microbiology, Oregon State University, 226 Nash Hall, Corvallis, OR 97331-3804, USA; ^2^Department of Biomedical Sciences, Oregon State University, 210 Dryden Hall, Corvallis, OR 97331-4801, USA; ^3^Department of Immunology, St. Jude Children's Research Hospital, 262 Danny Thomas Place, MS 351, Memphis, TN 38105-2794, USA

## Abstract

A cost-effective and efficacious influenza vaccine for use in commercial poultry farms would help protect against avian influenza outbreaks. Current influenza vaccines for poultry are expensive and subtype specific, and therefore there is an urgent need to develop a universal avian influenza vaccine. We have constructed a live bacterial vaccine against avian influenza by expressing a conserved peptide from the ectodomain of M2 antigen (M2e) on the surface of *Lactococcus lactis* (LL). Chickens were vaccinated intranasally with the lactococcal vaccine (LL-M2e) or subcutaneously with keyhole-limpet-hemocyanin conjugated M2e (KLH-M2e). Vaccinated and nonvaccinated birds were challenged with high pathogenic avian influenza virus A subtype H5N2. Birds vaccinated with LL-M2e or KLH-M2e had median survival times of 5.5 and 6.0 days, respectively, which were significantly longer than non-vaccinated birds (3.5 days). Birds vaccinated subcutaneously with KLH-M2e had a lower mean viral burden than either of the other two groups. However, there was a significant correlation between the time of survival and M2e-specific serum IgG. The results of these trials show that birds in both vaccinated groups had significantly (*P* < 0.05) higher median survival times than non-vaccinated birds and that this protection could be due to M2e-specific serum IgG.

## 1. Introduction

The US Poultry industry annually produces over 43 billion pounds of high-quality broiler chickens and turkeys and over 90 billion eggs, which in 2010 had a market value of $34.7 billion [1, 2]. Avian diseases are a constant threat to the industry. Viruses are of particular concern because antibiotics cannot control them, although vaccines can control some avian viral infections. Avian vaccines are an important component of protecting the value of commercial poultry. However, many commercial birds are not vaccinated because of the cost, labor, and difficulty in differentiating infected from vaccinated animals.

Avian influenza virus is an important concern to the poultry industry both in the USA and worldwide. It is highly contagious and causes two levels of disease [3]. Low pathogenic strains cause a disease that is seldom fatal but results in slower growth and lower egg production. The highly pathogenic form of the disease results in systemic morbidity and a high mortality rate (90–100%). Highly pathogenic avian influenza (HPAI) is a significant public health concern because of recent highly pathogenic H5N1 avian influenza outbreaks causing human deaths in Asia, Europe, Middle East, and Africa. According to the world health organization (WHO) update, since 2003 until February 2013, there were 620 confirmed cases of human infection with H5N1, of which 367 died due to disease complications. Although there are avian influenza vaccines approved in the USA for use in commercial poultry, they are subtype specific and costly to administer because they require parenteral delivery (intramuscular or subcutaneous).

*Lactococcus lactis* (LL) is a nonpathogenic, Gram-positive bacterium that is being developed as a delivery vehicle for vaccines. Various heterologous bacterial and viral antigens have been expressed from *L. lactis*, and antigen-specific immune responses have been reported [4–12]. The efficacy of lactococcal vaccines has been validated in many reports that have shown protection from infectious challenge of vaccinated animals [4, 12–15]. In mammals, *L. lactis* does not colonize the oral cavity or gastrointestinal tract but remains metabolically active and survives passage after oral administration [16–19]. It is thought that noncolonizing bacteria may be preferred over commensal bacteria for vaccine delivery because they may avoid antigen tolerance [20]. Little is known about *L. lactis* in chickens, but the closely related *Streptococcus* genus is abundant in chicken gastrointestinal contents [21, 22].

The M2 protein of avian influenza virus is one of three proteins with domains exposed outside the virus particle. The ectodomain of M2 (M2e) includes a peptide region that is conserved among all subtypes and therefore has been a main focus for the development of a universal influenza vaccine. In the intact virion, M2e is not the dominant immunogen [23–26]. However, antibodies to the M2e peptide increase survival and reduce disease upon infectious challenge in mice and chicken [27–32]. 

In this report, live *L. lactis* that expresses M2e (LL-M2e) or keyhole-limpet-hemocyanin- (KLH-) conjugated M2e (KLH-M2e) was used to vaccinate chickens. Immune responses were measured, and the vaccinated and nonvaccinated birds were challenged with highly pathogenic avian influenza virus. The results of these trials show that birds in both vaccinated groups had significantly (*P* < 0.05) higher median survival times than nonvaccinated birds and that this protection could be due to M2e-specific serum IgG. 

## 2. Material and Methods

### 2.1. Vaccine Construction

**Figure 1:**
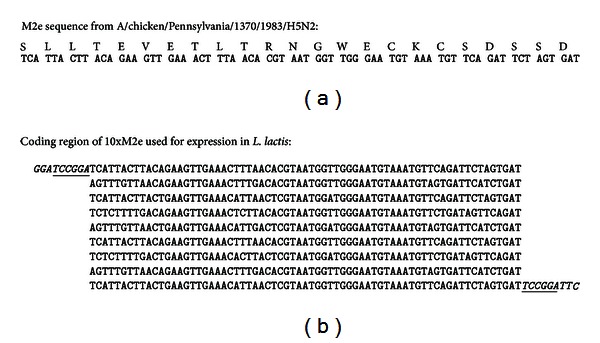
Coding region of 10xM2e used for expression in *L. lactis*. (a) Protein and nucleic acid sequence of M2e from A/chicken/Pennsylvania/1370/1983/H5N2 is shown. (b) Nucleic acid sequence of coding region of 10xM2e used for expression in *L. lactis* is shown. The bases in italics were added to the ends of the coding region for 10xM2e. *BspE1* sites (underlined) were used to clone the sequence in-frame into the unique *BspE1* site in pip.

DNA encoding (Figure 1) 10 tandem copies (10xM2e) of M2e (SLLTEVETLTRNGWECKCSDSSD) was prepared commercially (Blue Heron Biotechnology) using codons preferred by *L. lactis*. *BspE1* restriction sites were included at each end for cloning. 10xM2e was cloned into the lactococcal expression vector pP16pip as described [8] using standard methods, creating pBG-10xM2e, and transformed [33] into the plasmid-free strain *L. lactis* LM2301 [34].

### 2.2. Polyclonal Antiserum

M2e peptide was prepared commercially (Global Peptide Services) and covalently linked to keyhole limpet hemocyanin (KLH) using a commercial kit according to the manufacturer's instructions (Thermo-Fisher Scientific). Polyclonal antiserum was prepared by injecting subcutaneously New Zealand white rabbits with 50 *μ*g (M2e-equivalent) conjugate (KLH-M2e) mixed with adjuvant (TiterMax). M2e-specific titer was measured by ELISA as described [35]. 

### 2.3. *L. lactis* Surface Expression of M2e

M2e expression was measured using a modification of the indirect cellular ELISA [35]. Exponential phase cultures of *L. lactis* (pBG-10xM2e) were centrifuged (5,000 ×g, 5 min), washed, and resuspended in PBS to a final cell optical density (600 nm) = 1.0. Serial 1 : 2 dilutions of washed cells were added to a 96-well microtiter plate. Plates were further processed as described using polyclonal rabbit anti-M2e serum and preimmune serum, goat-anti-rabbit IgG-horseradish peroxidase conjugate (Santa Cruz Biotechnology), and chemiluminescent substrate (Thermo-Fisher Scientific). Plates were read in a Tecan Infinite F500 plate reader. 

### 2.4. Virus Stocks

Highly Pathogenic Avian influenza A/chicken/Pennsylvania/1370/1983 (H5N2) virus was obtained from United States Department of Agriculture. Viral stocks were grown in day 10 embryonated chicken eggs for 3–5 days. Allantoic fluid was collected, tested by hemagglutination assay [36], and stored at −85°C. Egg infectious dose (50%) was calculated by the formula of Reed and Muench [37].

### 2.5. Serum and Fecal Analysis

Serum and fecal samples were analyzed for M2e antibodies by ELISA [35] using microtiter plates coated with 2 *μ*g/mL M2e peptide. A standard curve was included on each plate by making a serial dilution of chicken IgG (Rockland). The plates were developed with goat antichicken IgG-horseradish peroxidase conjugate (Bethyl Laboratories) and chemiluminescent substrate (Thermo-Fisher Scientific). Luminescent signal was converted to IgG concentration by extrapolation from the graph of luminescence versus IgG concentration of the standards, and corrected for sample dilution. Each sample was analyzed at three dilutions in duplicate. 

### 2.6. Cell-Mediated Immune Response

A lymphocyte proliferation assay was performed as described [38]. Peripheral lymphocytes were isolated from blood collected 14 days after the final vaccination, stained with carboxyfluorescein diacetate succinimidyl ester, and stimulated for 72 h at 39°C with 20 *μ*g/mL M2e peptide, 20 *μ*g/mL nonspecific peptide, or 10 *μ*g/mL concanavalin A (Sigma). Stimulated and nonstimulated lymphocytes were stained with mouse antichicken CD4-R-phycoerythrin clone CT-4 (Thermo-Fisher Scientific) and analyzed by flow cytometry on a Cytomics FC 500 instrument (Becton Dickinson) and FlowJo software (Tree Star).

### 2.7. Vaccination and Challenge

Two groups of six seventeen-day-old Neo Brown chickens were vaccinated intranasally on three consecutive days with 4 × 10^10^ cfu LL-M2e in 100 *μ*L PBS. The regimen was repeated 2 and 4 weeks later. Six seventeen-day-old Neo Brown chickens were each vaccinated subcutaneously on the back of the neck with a 1 : 1 mixture of Titermax Gold adjuvant and 50 *μ*g M2e equivalent KLH-M2e conjugate in a total volume of 400 *μ*L. The subcutaneous vaccination was repeated 2 and 4 weeks later. A negative control group of six seventeen-day-old Neo Brown chickens was not vaccinated. *L. lactis* control was not used in the experiment because of two reasons: (1) previous data in our lab [8, 14] and previous publications have suggested that *L. lactis* does not elicit acquired immune response [4–15], and (2) the HEPA-filtered primary containment cage within the animal biosafety laboratory-3+ (ABSL-3+) could accommodate only 12 birds at a time and it was decided by investigators and Oregon State ethical committee members to use vaccinated and nonvaccinated groups only. Serum was collected before vaccination and 1 week after the last vaccination. Fecal samples were collected before and after vaccination, mixed with 0.3 mL 0.5% bovine serum albumin, 0.02% NaN_3_, and 1x protease inhibitor (Boehringer Mannheim) in PBS, and stored at −20°C until assayed. 

Birds were challenged intranasally 2 weeks after vaccination with 1 × 10^4^ egg infectious dose of highly pathogenic avian influenza virus A/chicken/Pennsylvania/1370/1983 (H5N2) in 100 *μ*L PBS using a micropipettor to deliver the virus into the nasal opening. Tracheal swabs were collected prior to infection and on day 3 after infection. Body weight and cloacal temperature were recorded daily.

### 2.8. Tracheal Swab Analysis

Tracheal swabs were collected by inserting a calcium alginate fiber-tipped applicator swab (Fisher Scientific) into the trachea and moving the swab 10 times up and down about 1 cm. The swab was immediately placed in 1 mL minimal essential medium (Life Technologies) plus streptomycin (100 *μ*g/mL), penicillin (100 units/mL), and amphotericin B (0.25 *μ*g/mL) and stored at −80°C. Viral content of the tracheal swabs was assessed by plaque assay. Briefly, the swabs were thawed and mixed by vortexing. A 1 : 10 dilution series of each sample was prepared in PBS and then added to tissue cultures of Madin-Darby canine kidney cells (MDCK). After 72 h at 37°C + 5% CO_2_, plates were fixed with formalin and stained with crystal violet, and plaques were counted.

### 2.9. Statistical Analysis

Data were analyzed using GraphPad software, Prism 4.0, and InStat 3.0. Serum means and tracheal plaques were compared using two-tailed, unpaired Student's *t*-test with Welch's correction. Correlation analysis between serum response and survival was done using Pearson's analysis (two-tailed), and a line was plotted using linear regression analysis. Survival was analyzed by the method of Kaplan and Meier using the logrank test. Temperature and weight change was analyzed using nonparametric ANOVA (Kruskal-Wallis test).

### 2.10. Animals

All procedures using animals complied with all state and federal laws and were approved by the Oregon State University Institutional Animal Care and Use Committee (approval number 3682). All experiments involving high pathogenic avian influenza virus were conducted in CDC/APHIS-USDA approved ABSL-3+ high containment facility at VMAIL, Oregon State University.

## 3. Results

### 3.1. Vaccine Design and Construction

The M2e sequence from HPAI strain A/Chicken/Pennsylvania/1370/1983/H5N2 was selected for construction of the vaccine (Figure 1). Ten tandem repeats of the coding region for the M2e were cloned in-frame into the coding region for a surface protein from *L. lactis*. The genetic fusion was cloned into an expression vector with a strong promoter for *L. lactis* and transformed into *L. lactis* (LL-M2e).

### 3.2. Expression of M2e

**Figure 2:**
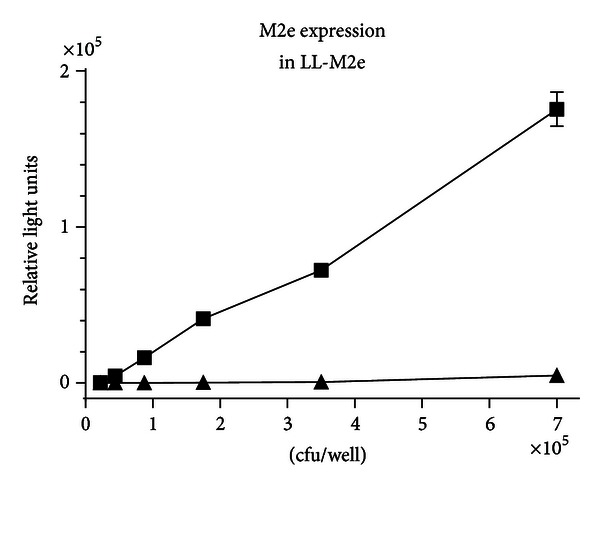
Expression of M2e from *L. lactis*. A 1/2 serial dilution of LL-M2e washed cells was bound to a 96-well microtiter plate and analyzed by ELISA. Cell surface-bound M2e was detected by adding a constant amount/well of preimmune (▲) or postimmune (■) rabbit polyclonal M2e antiserum. Expression of M2e protein on the surface of LL vector with no M2e gene was not detected using preimmune and polyclonal antibodies (data not shown). The signal was developed with secondary antibody anti-rabbit IgG-horseradish peroxidase conjugate and chemiluminescent substrate. Error bars indicate standard deviation. Three independent experiments were conducted (*n* = 3).

Expression of M2e protein on the surface of LL-M2e was measured by ELISA assay using preimmune and rabbit polyclonal anti-M2e antibodies. The results show that the level of expression of M2e was proportional to the amount of LL-M2e bound to the ELISA plate (Figure 2). Preimmune serum did not show any signal. The expression of M2e protein on the surface of LL vector with no M2e gene was not detected using preimmune and polyclonal antibodies suggesting that the antibody specific to M2e is not binding to any protein from the LL vector with no M2e gene (data not shown). Three independent experiments were conducted.

### 3.3. Immune Response

Intranasal vaccination with LL-M2e was tested in 12 chickens. In addition, another group of 6 chickens was vaccinated subcutaneously with M2e peptide conjugated to keyhole limpet hemocyanin (KLH-M2e). Six birds were nonvaccinated, which formed a negative control group. One week after the final dose of vaccine, blood was collected, and the M2e-specific serum IgG response was measured by ELISA.

**Figure 3:**
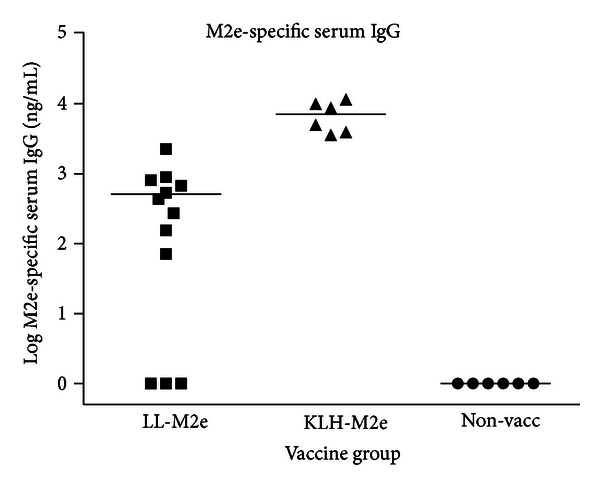
Serum response. Serum was collected 1 week after final vaccination and analyzed for M2e-specific serum IgG by ELISA using microtiter plates coated with 2 *μ*g/mL M2e peptide. The plates were developed with goat antichicken IgG-horseradish peroxidase conjugate and chemiluminescent substrate. Luminescent signal from each sample was converted to concentration of chicken IgG by extrapolation from the graph of luminescence versus IgG concentration of the standards and corrected for sample dilution. Each sample was analyzed at two or three different dilutions, and each dilution was analyzed in duplicate. Group mean M2e-specific IgG concentration is shown.

The results indicate that 8 of 12 birds vaccinated with LL-M2e had a measurable humoral response, and the group mean was significantly (*P* < 0.05) higher than that of the nonvaccinated group (Figure 3). All birds vaccinated with KLH-M2e had a measurable M2e-specific response that was significantly (*P* < 0.05) higher than responses in either of the other groups. None of the birds in the nonvaccinated group showed an M2e-specific humoral response.

### 3.4. Infectious Challenge

Two weeks after the final dose of vaccine, the vaccinated (LL-M2e and KLH-M2e vaccination groups) and nonvaccinated chickens were challenged with the highly pathogenic strain A/chicken/Pennsylvania/1370/H5N2. 

**Figure 4:**
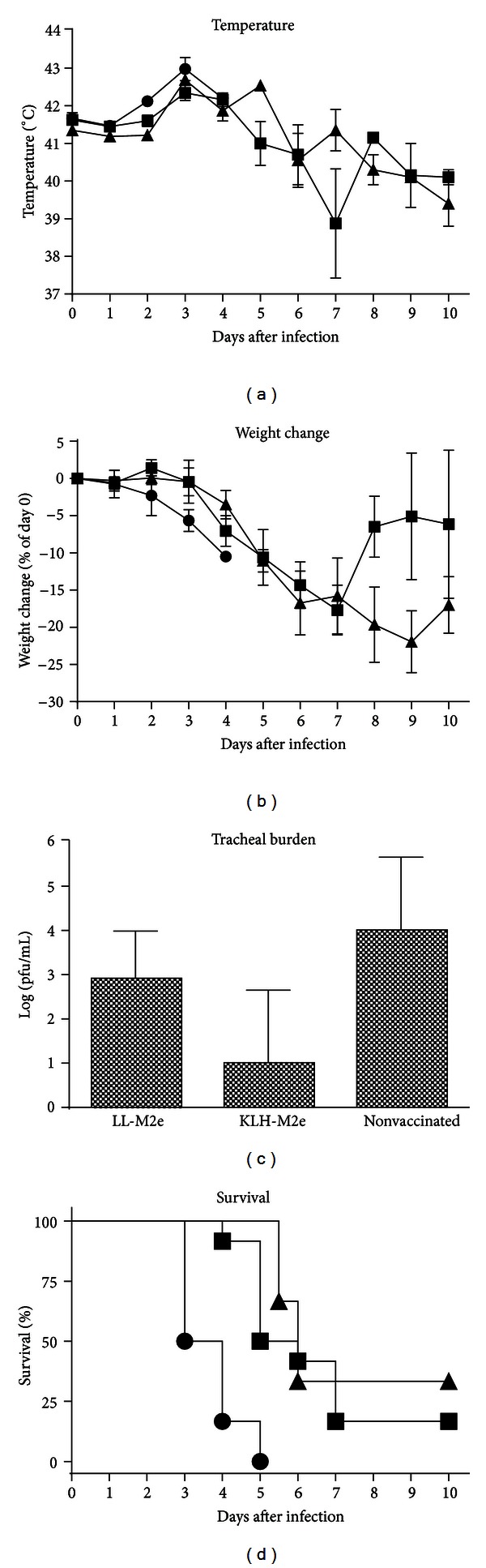
Weight loss, body temperature, tracheal burden, and survival. Groups of vaccinated (intranasal LL-M2e (■), subcutaneous KLH-M2e (▲)) or nonvaccinated (*⚫*) birds were challenged with high pathogenic avian influenza H5N2 (A/chicken/Pennsylvania/1370/1983). *n* = 12 (LL-M2e), *n* = 6 (KLH-M2e and nonvaccinated). Error bars indicate standard deviation. (a) Body temperature was measured for each bird, and the group mean is shown. (b) Weight loss for each bird was calculated as a difference compared to day 0, and the group mean is shown. (c) Tracheal swabs were collected 3 days after infection and analyzed for viral plaques. (d) Survival was monitored for 10 days after infection.

Body temperature was monitored, and the results show that all groups responded similarly (Figure 4(a)). Temperatures rose with an average of 1.04°C from day 0 to day 3 and then declined with an average of 1.38°C by day 10 after infection. There was no statistically significant difference in temperatures among all groups at any time after infection.

Weight decreased to a maximum loss of between 18 and 22% over the first 7 to 9 days and then gradually increased (Figure 4(b)). There was no statistical difference in weight loss among the groups at any time after infection.

Viral burden in tracheal swabs was measured 3 days after infection (Figure 4(c)). Mean tracheal burden varied among groups from 10^1^ to 10^4^ pfu/mL, and there was a significantly (*P* < 0.05) lower mean value for birds vaccinated with KLH-M2e than either of the other two groups. There was no significant (*P* > 0.05) difference between the group vaccinated with LL-M2e and the nonvaccinated group. 

Birds in both vaccinated groups had significantly (*P* < 0.05) higher median survival times than nonvaccinated birds (Figure 4(d)). Birds vaccinated with LL-M2e or KLH-M2e had median survival times of 5.5 and 6.0 days, respectively. Nonvaccinated birds had a median survival time of 3.5 days. Two of 12 birds from the LL-M2e group survived and 2 of 6 birds from the KLH-M2e group survived, whereas none of the six nonvaccinated birds survived. There was no statistical difference in survival between the two vaccinated groups.

**Figure 5:**
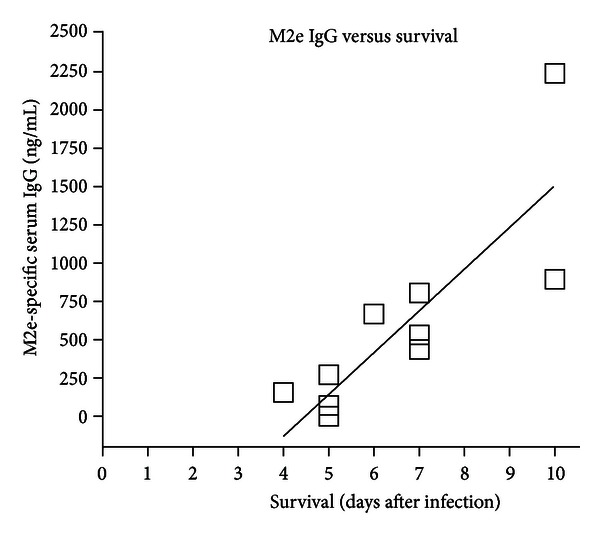
Correlation analysis. M2e-specific serum IgG from birds vaccinated with LL-M2e (*n* = 12) was plotted as a function of survival following infectious challenge. The line represents a linear regression analysis.

An analysis of M2e-specific serum IgG as a function of survival showed a significant (*P* < 0.01, *R* ^2^ = 0.9197) correlation for the group vaccinated with LL-M2e (Figure 5). An analysis of the group vaccinated with KLH-M2e showed a similar trend, but the correlation was not statistically significant (*P* > 0.05, data not shown).

Although there was a correlation between M2e-specific serum response and survival in the group vaccinated with LL-M2e, additional immune responses were analyzed. CD4^+^ T lymphocytes from 6 LL-M2e-vaccinated birds were analyzed for M2e-specific proliferation. In addition, fecal samples for detection of IgA were collected 2 weeks after final vaccination and just prior to infection. In all of the birds tested, M2e-specific CD4^+^ lymphocyte response or fecal IgA was below a threshold limit of our assay in vaccinated birds (data not shown). 

## 4. Discussion

The ectodomain of the M2 protein is an attractive choice for a cross-subtype vaccine. Its amino acid sequence is not only highly conserved, but also nonglycosylated, which is essential for antigens expressed from bacteria. Although M2e is only weakly antigenic in the context of an infection or whole virus vaccine, antibodies against M2e reduce infection and protect against viral replication and death, at least in mammalian models [25, 28–32, 39].

The strategy of using M2e as a vaccine is different from the conventional one currently used for human seasonal vaccines. Current vaccines and natural infection induce a humoral response to two immunodominant viral surface antigens, hemagglutinin (HA) and neuraminidase (NA). However, HA and NA undergo intense selective pressure due to the host immune response and are constantly changing. The use of M2e as antigen would avoid the problem of genetic drift and shift that characterizes both HA and NA because M2e is genetically stable. Even under prolonged selective pressure, only a single amino acid (proline to leucine or histidine at position 9) has been found to change in M2e [40]. It is likely that the genetic drift in the highly conserved M2e would be low [26, 41], which suggests that M2e vaccines would be universally effective against many subtypes and would not require seasonal modification like the current influenza vaccines. 

*L. lactis* has been used as a vaccine delivery vehicle for various types of antigens, including those from bacteria and viruses [7, 13, 25, 42, 43]. Our expression system in *L. lactis* has been used previously to display an antigen (M6c) from *Streptococcus pyogenes* [8]. Mice vaccinated intranasally with the *L. lactis*-M6c developed a significant humoral response to M6c that correlated with protection from infectious challenge [14].

In the present report, we have similarly expressed 10 tandem copies of M2e on the surface of *L. lactis* and measured immune responses. Most of the vaccinated chickens developed an M2e-specific serum IgG response. The lack of robust M2e-specific fecal IgA or CD4^+^ T lymphocyte response could be due to differential processing of antigen by the immune system, which needs to be explored further. This indicates that LL-M2e induced mainly humoral response, but less significant cellular response.

The infectious challenge results show that chickens vaccinated intranasally with LL-M2e or vaccinated subcutaneously with KLH-M2e survived infectious challenge longer than nonvaccinated birds. Birds vaccinated with LL-M2e or KLH-M2e had median survival times of 5.5 and 6.0 days, respectively. Nonvaccinated birds had a median survival time of 3.5 days. Two of 12 birds from the LL-M2e group survived and 2 of 6 birds from the KLH-M2e group survived, whereas none of the six nonvaccinated birds survived. Birds in both vaccinated groups had significantly (*P* < 0.05) higher median survival times than nonvaccinated birds.

Weight loss or body temperature did not differ much among treatment groups, and therefore we believe that apparently these measures of health were not predictive of survival. 

Viral burden was also measured. Previous experiments (not shown) indicated that viral burden in the tracheal swabs peaked at day 3 after infection. Birds vaccinated subcutaneously with KLH-M2e had a lower mean viral burden than either of the other two groups. Perhaps this is a reflection of the higher M2e-specific serum IgG. We found no statistical significance in the amount of virus in tracheal swabs at day 3 after infection between birds vaccinated with LL-M2e and nonvaccinated birds. 

An analysis of our data showed that protection may be due to M2e-specific serum IgG. Birds with higher M2e-specific IgG tended to survive longer. Previous studies present conflicting results on the mechanism of protection provided by M2e vaccines. Some studies show that antibodies to M2e provide protection [32, 44, 45]. Another study showed no correlation between M2e-specific titer and protection from infectious challenge [46]. Some reports show that M2e vaccines can induce an M2e-specific CD4^+^ T-cell response that may contribute to protection [47, 48]. Still other reports suggest that M2e-specific antibodies may bind to infected cells and direct natural killer T cells, macrophages, or other host immune cells to kill the infected cell [26, 49–51]. A recent article by El Bakkouri et al. [52] has suggested that alveolar macrophages and Fc receptor-dependent elimination of influenza A virus-infected cells are essential for immune protection by anti-M2e IgG. Our results are consistent with a mechanism of protection that depends on an M2e-specific serum IgG response. 

## 5. Conclusion

In conclusion, the data show that intranasal vaccination of chickens with LL-M2e or subcutaneous vaccination of chickens with KLH-M2e provided a significant increase in survival compared to nonvaccinated birds. Survival and protection could be due to serum M2e-specific IgG response to the vaccine.
